# Evaluating algorithms of decision tree, support vector machine and regression for anode side catalyst data in proton exchange membrane water electrolysis

**DOI:** 10.1038/s41598-023-47174-w

**Published:** 2023-11-20

**Authors:** Mahdi Arjmandi, Moslem Fattahi, Mohsen Motevassel, Hosna Rezaveisi

**Affiliations:** 1 Chemical Engineering Department, Abadan Faculty of Petroleum Engineering, Petroleum University of Technology, Abadan, Iran; 2 Department of Chemical and Materials Engineering, University of Alberta, Edmonton, AB Canada; 3 Chemical Engineering Department, Faculty of Engineering, Razi University, Kermanshah, Iran

**Keywords:** Mathematics and computing, Scientific data, Chemical engineering

## Abstract

Nowadays, due to the various type of problems stemmed from using chemical compounds and fossil fuels which have widely influence on whole environment including acid rain, polar ice melting and etc., number of researches have been leading on replacing the nonrenewable energy sources with renewable ones in order to produce clean fuels. Among these, hydrogen emerges as a quintessential clean fuel, garnering substantial attention for its potential to be synthesized from the electric power generated by renewable sources like nuclear and solar energies. This is achieved through the employment of a proton exchange membrane water electrolysis (PEMWE) system, widely recognized as one of the most proficient and economically viable technologies for effecting the separation of H_2_O into H^+^ and OH^−^. In this study, the important affecting parameters on the anode side of catalyst in PEMWE and analyzed them by machine-learning (ML) algorithms through developing a data science (DS) procedure were discussed. Various machine learning models were subjected to comparison, wherein the Decision Tree models, specifically those configured with maximum depths of 3 and 4, emerged as the optimal choices, attaining a perfect 100% accuracy across both Dataset 1 and Dataset 2. Moreover, notable enhancements in accuracy values were observed for the Support Vector Machine (SVM) model, registering increments from 0.79 to 0.82 for Dataset 1 and 2, respectively. In stark contrast, the remaining models experienced a decrement in their accuracy scores. This phenomenon underscores the pivotal role played by the data generation process in rendering the models more faithful to real-world scenarios.

## Introduction

In recent years, concerns about global warming and its various environmental impacts, such as polar ice melting, acid rain, and rising sea levels, have become a primary focus for scientists. These issues are largely attributed to the consumption of refractory chemical compounds, particularly fossil fuels like coal, oil, and natural gas, as well as concerns about their depletion^1^. Consequently, there has been a concerted endeavor to expedite the advancement of renewable energy generation, storage, and conversion infrastructures, in light of projections indicating a prospective global power demand of approximately 30 and 46 TW by the years 2050 and 2100, respectively^2^. However, a major challenge in using solar and wind energy as renewable sources is their unscheduled and intermittent supply, which often does not match the grid power demands^3^. To address this issue, efficient systems for storing excess electricity must be developed. One promising approach is the use of electrocatalytic systems, which convert electricity into chemical energy for indirect storage of excess renewable energy^4^. Amongst a plethora of electrocatalytic technologies, water electrolysis stands out as the most efficacious means for producing pristine green hydrogen, harnessing the potential of renewable energy sources like solar and wind energies^5^. Pertinently, Pourrahmani et al. have undertaken a thorough inquiry into the feasibility and efficacy of employing PEMFC as an indirect means of water electrolysis storage, a process entailing the conversion of excess electricity generated by wind turbines into hydrogen gas^6^. This green hydrogen can be stored and used in the chemical industry, or for electricity production via fuel cells or internal combustion engines, with zero post-combustion pollutants^7^. Commercially available systems for water electrolysis include alkaline, solid oxide and proton exchange membrane (PEM) electrolyzers, with the latter being more advantageous due to their more compact design, absence of leaking issues, higher current density, higher operating temperature and characteristic of high temperature potential resulting in higher energy conversion efficiency, greater hydrogen generation rate, lower gas crossover rate resulting in decreasing power consumption, part-load operating ability, and ability to operate at higher pressures due to their strong cell structure^8–11^. In contemporary times, there exists a burgeoning inclination towards the refinement and reconfiguration of alkaline water electrolyzers, coupled with the advancement of proton exchange membranes with applicability spanning both water electrolysis units and fuel cells. These strides stem from notable advancements witnessed within the domain of high-temperature solid oxide technology, as substantiated by research studies^12–14^.

Water electrolysis is a process that water molecules spilt in hydrogen and oxygen gases using electricity through electrochemical process resulting in producing clean energy with no emission of pollution. The basic equation of water electrolysis is as Eq. 1^15^.

1 $$H_{2} O\left( {liquid} \right) + Energy \to H_{2} \left( g \right) + {\raise0.7ex\hbox{$1$} \!\mathord{\left/ {\vphantom {1 2}}\right.\kern-0pt} \!\lower0.7ex\hbox{$2$}}O_{2} \left( g \right)$$

**Figure 1:**
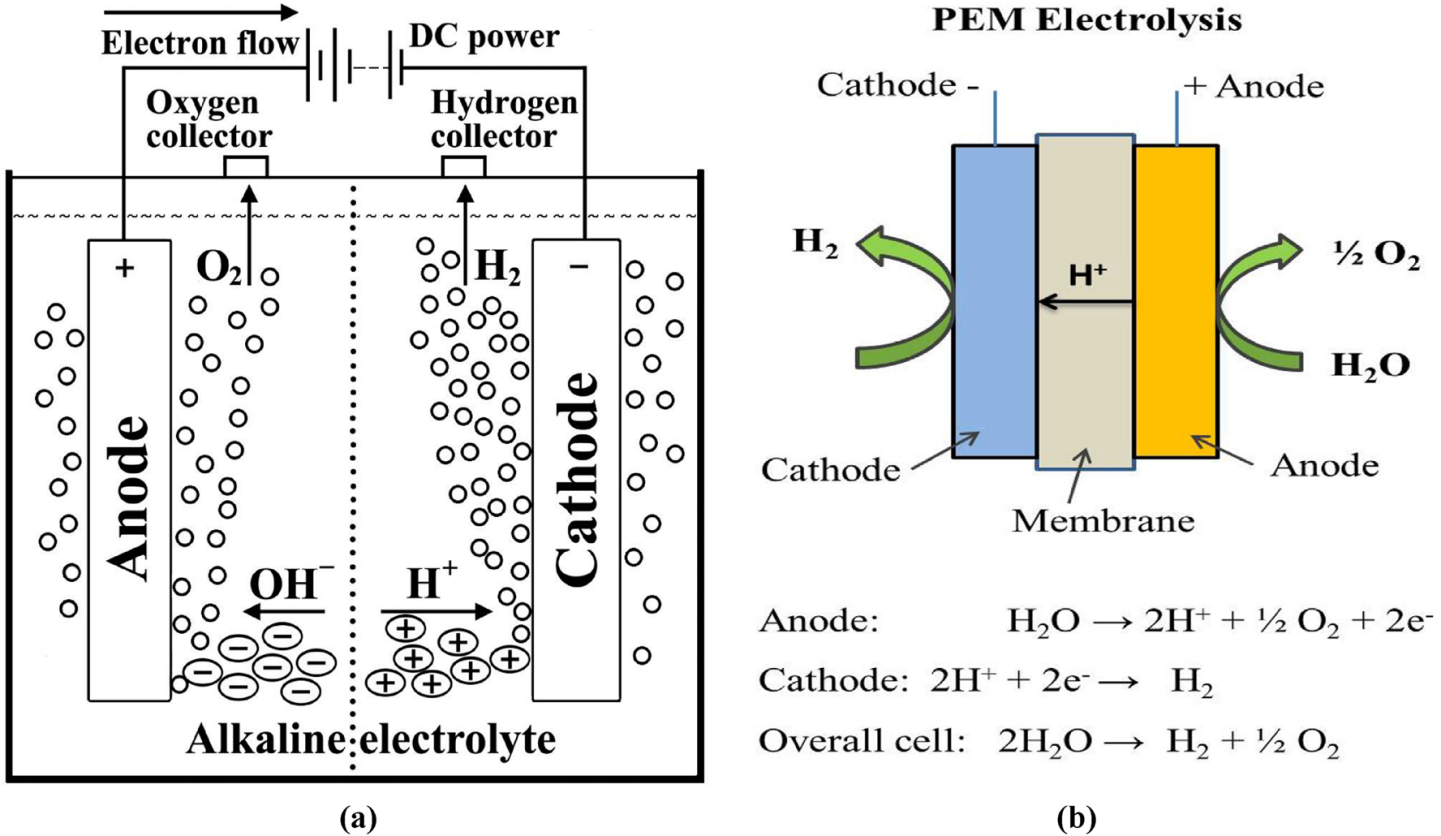
**(a)** Scheme principle for electrolysis cell^14^ and **(b)** PEM water electrolysis.

The simplest water electrolysis system has been displays in Fig. 1a which consisting of an anode and a cathode connected through an external power supply and immersed in a conducting electrolyte. Through the imposition of a direct current (DC) upon the system, electrons traverse from the negative terminal of the DC power source towards the cathode. Here, they are absorbed by hydrogen ions (protons), thereby engendering the formation of hydrogen atoms. In the overarching scheme of water electrolysis, hydroxide and hydrogen ions migrate towards the anode and cathode respectively, a diaphragm serving as a delineating barrier between these two segments. Additionally, the hydrogen and oxygen produced at the cathode and anode, respectively, are captured by gas collectors^14^.

Several electrolyte systems have been developed for water electrolysis, including alkaline water electrolysis (AWE), proton exchange membranes (PEMs), alkaline anion exchange membranes (AEMs), and solid oxide water electrolysis (SOE). Despite the utilization of diverse materials and operational parameters, these systems adhere to a common set of fundamental principles. Moreover, water electrolysis can be conducted across a spectrum of temperatures, contingent on the specific operational criteria and temperature range selected^16^.

Within the realm of designing experiments for cells, a pivotal stage involves scrutinizing the experimental data to discern the optimal values for an array of parameters influencing cell performance. In this endeavor, the novel concepts of Artificial Intelligence (AI), Internet of Things (IoT), Data Science (DS), and Machine Learning (ML) emerge as relatively recent paradigms. They hold the potential to enhance the efficiency of fuel cells and augment hydrogen generation through the assimilation of historical data and predicted futures^17,18^. This study centered on examining the introduction and discussion of proton exchange membrane (PEM) electrolyzers, with a specific focus on integrating data science and machine learning principles. In pursuit of this goal, a data science procedure was devised, utilizing machine-learning algorithms and incorporating anode side catalyst parameters. The outcomes of this analysis were subsequently scrutinized using Jupyter notebook, a programming platform utilizing Python 3.9.0 facilitated by the Anaconda platform. Finally, the various models were compared, and the resulting data were visualized based on different values of model evaluation parameters which resulted in realizing the application of data science as an auxiliary tool in analyzing the data and obtaining practical models for predicting and discovering optimal data based on the changes of other influencing experimental parameters related to the anode side of the catalyst in the production of hydrogen gas through PEM water electrolyzer.

## PEM water electrolyzer (PEMWE)

The first PEM water electrolysis named as polymer electrolyte membranes or solid polymer electrolyte membranes in 1966 for U.S space applications which was related with sulfonated polystyrene ion-exchange electrolyte membrane development idealized by Grubb in 1950s to solve the problems of the alkaline water electrolysis^19,20^. In most of the PEM water electrolyzer, perfluorosulfonic acid membranes such as Nafion® and sulfonated polyetheretherketone have used as an electrolyte (proton conductor)^21,22^. These proton exchange membranes having many advantages such as lower gas permeability, high proton conductivity (0.1 ± 0.02 S cm^−1^), lower thickness (∑20–300 µm) and high-pressure operations. PEM water electrolysis is one of the promising methods for conversion of renewable energy to high pure hydrogen in terms of sustainability and environmental influence. Proton exchange membrane water electrolysis also offers several advantages, such as a compact design, high current density exceeding 2 A.cm^−2^, high efficiency, fast response, small footprint, and operation under lower temperatures ranging from 20–80 °C. Moreover, it produces ultrapure hydrogen and oxygen as a byproduct^21,23–26^. Notably, the process of balancing PEM electrolysis plants is relatively simple, which enhances its attractiveness for industrial applications.

The primary process of a PEM water electrolyzer involves the electrochemical splitting of water into hydrogen and oxygen at the cathode and anode sides, respectively. Specifically, water is introduced to the anode side, where an OER takes place, generating oxygen (O_2_), protons (H +), and electrons (e-). The electrons exit from the anode through the external power circuit, which provides the driving force (cell voltage) for the reaction. The protons that are produced travel to the cathode side through a proton-conducting membrane, resulting in a hydrogen evolution reaction (HER) that combines with the electrons to produce hydrogen, as depicted in Fig. 1b^27^.

The anode catalyst in proton PEMWEs has been the subject of extensive research due to the oxygen evolution reaction (OER) being the primary source of irreversibility^28^. Typically, noble metal-based electrocatalysts such as IrO_2_ are utilized for the OER in PEM water electrolysis, as it is recognized as one of the most durable materials under O_2_ evolution conditions in highly acidic environments^22,29^. However, this results in a higher cost compared to alkaline water electrolysis systems. Therefore, reducing production costs while maintaining high efficiency remains a significant challenge in PEM water electrolysis^22^.

## Data science and machine learning

### Data science theory

Data science often refers to the process of leveraging modern machine learning techniques to identify insights from data^30,31^. Over the past few years, there has been a growing trend among organizations to adopt a "data centered" approach to decision-making. As a result, there has been an increase in the formation of teams consisting of data science workers who collaborate on larger datasets, more structured code pipelines, and more consequential decisions and products^32^.

The demand for advanced data analytics leading to the use of machine learning and other emerging techniques can be attributed to the advent and subsequent development of technologies such as Big Data, business Intelligence, and the applications that require automation. Sandhu^33^ elucidates that machine learning is a subfield of artificial intelligence that employs computerized techniques to address problems based on historical data and information, without the need for significant modifications to the core process. Artificial intelligence, on the other hand, involves the development of algorithms and other computational techniques that imbue machines with intelligence. It comprises algorithms that can reason, act, and execute tasks using protocols that are beyond the capabilities of humans.

### Machine learning theory

Machine learning is a component of artificial intelligence although it endeavors to solve problems based on historical or previous examples^34^. Unlike artificial intelligence applications, machine learning involves learning of hidden patterns within the data (data mining) and subsequently using the patterns to classify or predict an event related to the problem^35^. Simply, intelligent machines depend on knowledge to sustain their functionalities and machine learning offers such a knowledge. In essence, machine learning algorithms are embedded into machines and data streams provided so that knowledge and information are extracted and fed into the system for faster and efficient management of processes. It suffices to mention that all machine learning algorithms are also artificial intelligence techniques although not all artificial intelligence methods qualify as machine learning algorithms.

Machine learning algorithms can be broadly classified as either supervised or unsupervised, although some authors may also include reinforcement learning as a distinct category, as these techniques involve learning from data to identify patterns with the goal of reacting to an environment. Nevertheless, most literature acknowledges the two major categories of supervised and unsupervised learning algorithms. The difference between these two main classes is the existence of labels in the training data subset. As outlined by Kotsiantis^36^, supervised machine learning involves the utilization of predetermined output attributes in conjunction with input attributes. These algorithms strive to predict and classify the predetermined attribute, and their performance is evaluated based on metrics such as accuracy, misclassification rate, and other relevant performance measures, which are contingent on the number of correctly predicted or classified instances of the predetermined attribute. Importantly, the learning process concludes when the algorithm attains a satisfactory level of performance^37^. According to Libbrecht and Noble^34^, technically, supervised algorithms perform analytical tasks first using the training data and subsequently construct contingent functions for mapping new instance of the attribute. As stated previously, the algorithms require prespecifications of maximum settings for the desired outcome and performance levels^34,37^. Machine learning methods typically require a training subset of around 66% of the data in order to achieve satisfactory results without incurring excessive computational costs^38^. Within the supervised learning paradigm, algorithms can be categorized into either classification or regression algorithms^35,36^. In contrast, unsupervised learning does not involve a target attribute and instead focuses on pattern recognition. All variables in the analysis are used as inputs, making these techniques particularly useful for clustering and association mining. According to Hofmann^39^, unsupervised learning algorithms are suitable for creating the labels in the data that are subsequently used to implement supervised learning tasks. That is, unsupervised clustering algorithms identify inherent groupings within the unlabeled data and subsequently assign label to each data value^38,40^. On the other hand, unsupervised association mining algorithms tend to identify rules that accurately represent relationships between attributes. Praveena^41^ asserts that supervised learning relies on prior experience or acquired patterns within the data and typically involves a defined output variable^42–46^. The input dataset is partitioned into train and test subsets, and various studies have explored the concept of training datasets based on the desired outcome^47–49^. Algorithms employing supervised learning utilize patterns within the training dataset to predict or classify an attribute within the test subset^50,51^. Multiple authors have described the workflow of supervised machine learning, and decision trees, Naïve Bayes, and Support Vector Machines are among the most commonly used algorithms^40,52–55^.

### Tools and systems

There exists a plethora of tools that are designed to support the work of data scientists. These include programming languages like Python or R, statistical analysis tools such as SAS^56^ and SPSS^57^, integrated development environments (IDEs) like Jupyter Notebook^58,59^, and automated model building systems such as AutoML^60^ and AutoAI^61^. Empirical studies have shed light on how data scientists utilize these tools^62–64^, as well as the features that could be augmented to enhance the user experience for those working solo^65^.

Jupyter Notebook^66^ is a noteworthy system, which has various versions including Google Colab^67^ and Jupyter-Lab^68^. It is an integrated development environment that is specifically tailored to meet the needs of data science workflows. The graphical user interface of Jupyter Notebook supports three core functionalities, which are vital to data science work: coding, documenting a narrative, and observing execution results^69^. Additionally, the capability to effortlessly switch between code and output cells enables data scientists to rapidly iterate on their model development and testing processes^31,62^.

## Developing data science procedure

### Data mining

**Figure 2:**
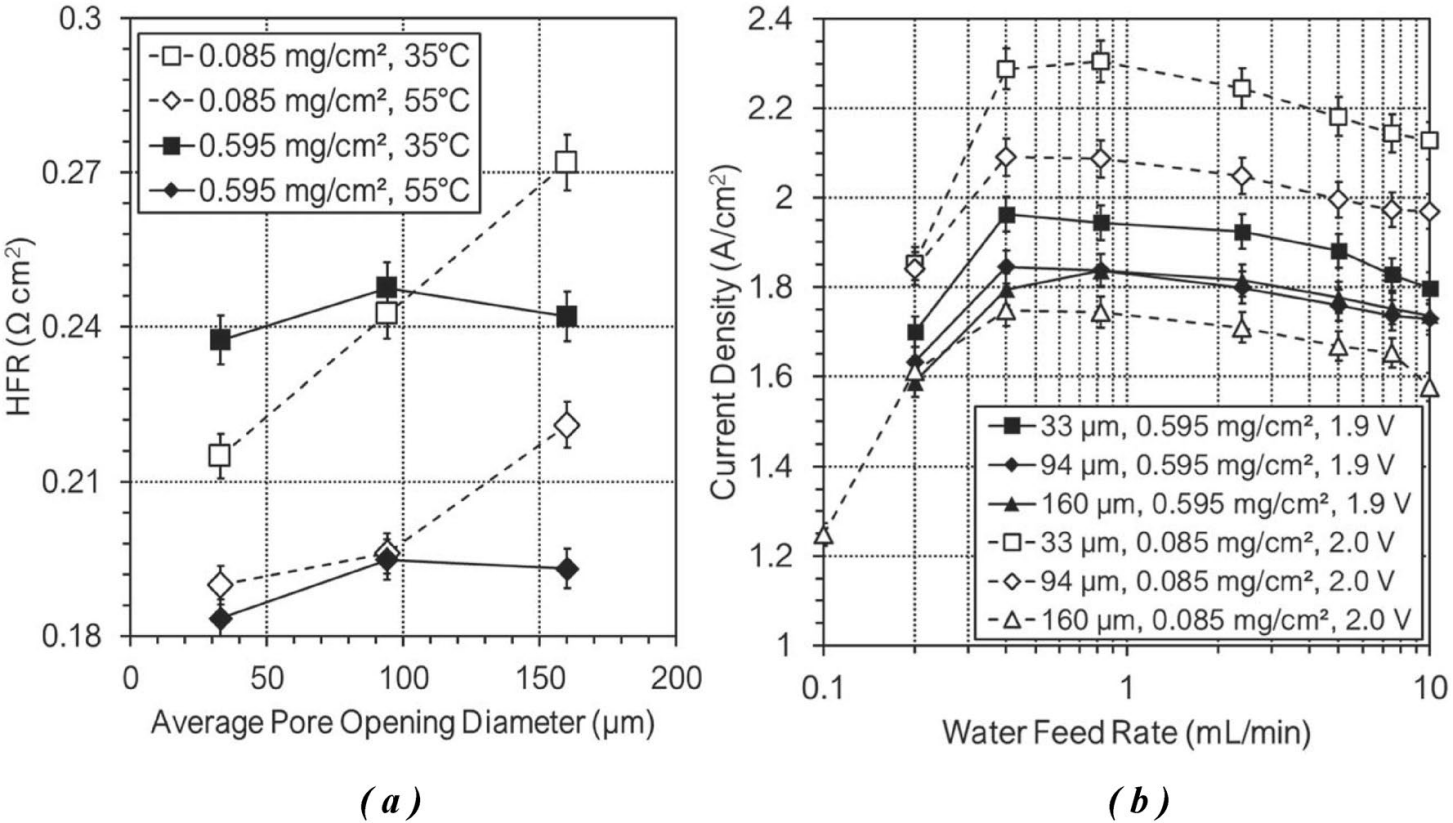
(**a**) trend in the HFR of the cell at 1.48 V by increasing PTL average pore opening diameter base on the IrO_2_ dosage changes and (**b**) current density vs the anode water feed rate for the 4 cm^2^ PEMWE cell under potentiostatic control at 55 °C different PTL average pore opening diameter base on the IrO_2_ dosage changes^70^.

This study takes into account the current density (CD), water feed rate (WFR), catalyst loading, and high-frequency resistance (HFR) of the anode side of a proton exchange membrane water electrolysis (PEMWE) system to develop a data science procedure and train machine learning models. The datasets were obtained from Fig. 2, which displays HFR vs. average pore opening diameter (APOD) at working temperatures of 35 °C and 55 °C, and CD vs. WFR at working cell potentials of 1.9 V and 2 V at 55 °C, respectively, based on two different catalyst loading levels of 0.595 and 0.085, along with the porous transport layer (PTL) material specifications of the anode side as the target data in the machine learning models. It should be noted that the datasets collected from Fig. 2a were only taken at 55 °C due to the temperature constraints of the CD vs. WFR experiment.

Three PTL cases have been investigated based on the average pore opening diameter, average grain diameter, areal surface porosity, average porosity and permeability as properties of these materials which are displays in Table 1.

**Table 1:** Properties of PTL materials^70^.

PTL	APOD (µm)	Avg. grain dia. (µm)	Areal surface por.	Avg. por.	Permeability (m^2^)
1	33	13.9	0.780	0.302	5.35e-13
2	94	30.7	0.730	0.312	1.10e-12
3	160	66.8	0.815	0.218	3.20e-13

### Data generation

The datasets mentioned above were initially processed and organized using MS Excel in preparation for modeling in Python Jupyter Notebook. The Dataset-1 consisted of 42 rows and 5 columns, but this was expanded to 162 rows and 5 columns by generating random numbers within a specific range for the WFR and CD columns using the code provided below:
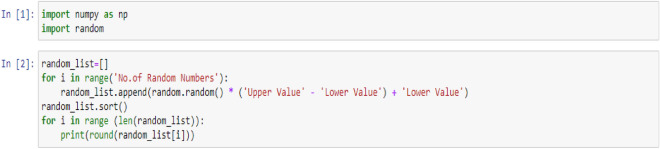


The generated data have been reentered into MS Excel for sorting and initial preparing which finally has been named Dataset-2.

### Input variables

In the subsequent phase, three discrete machine learning models—Regression, Support Vector Machine (SVM), and Decision Tree—will be individually employed on Dataset-1 and Dataset-2. The resultant shifts in WFR and CD concerning the PTL will be displayed in dedicated visual representations, owing to the marked distinctions in their respective datasets. To effectuate this, each dataset has been imported into Python Jupyter Notebook using the prescribed code as presented below:
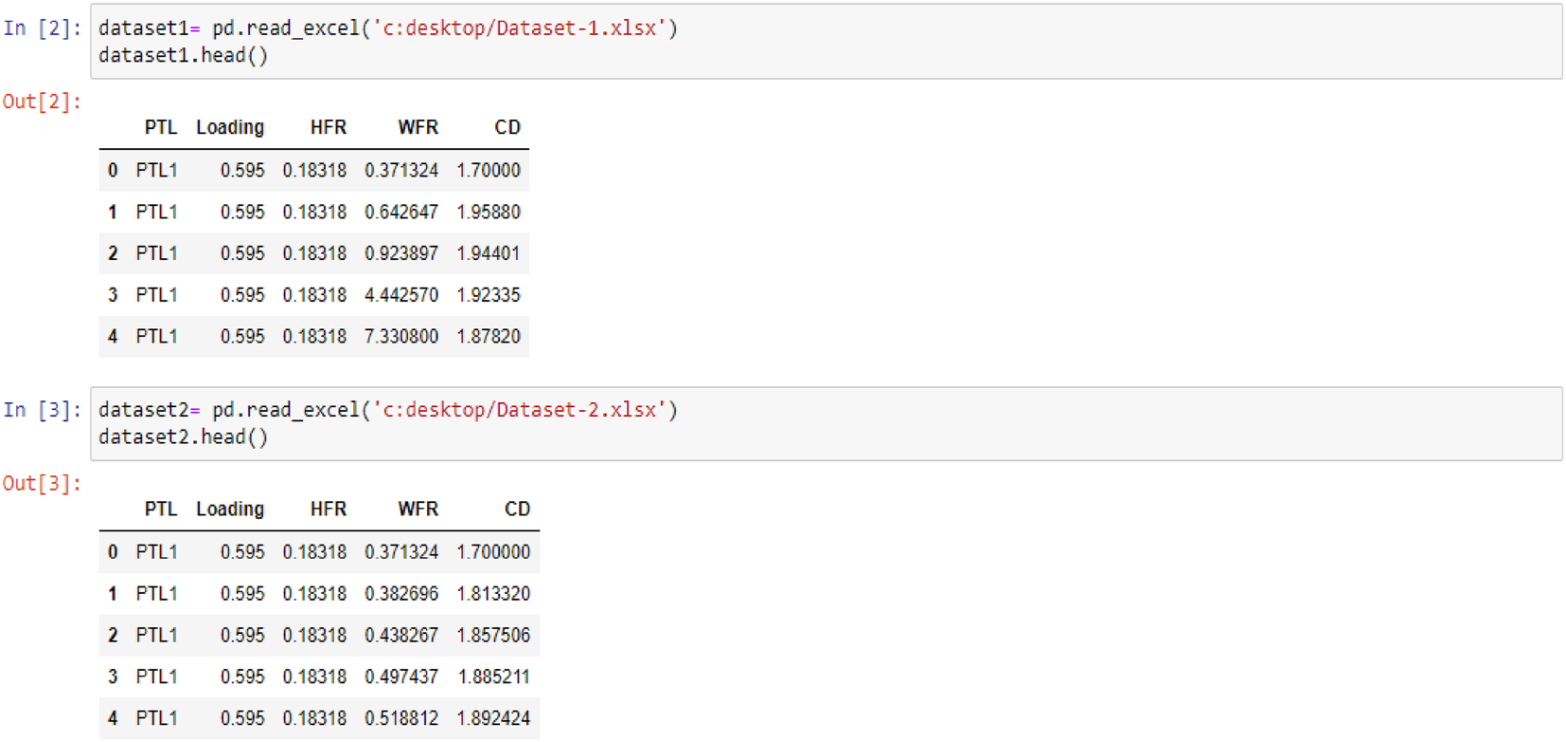


### Data pre-processing

The machine learning models will not work correctly in presence of the letters, so the words PTL1, PTL2 and PTL3 were replaced with a series of indexes including the numbers 0, 1 and 2 respectively by the following code:
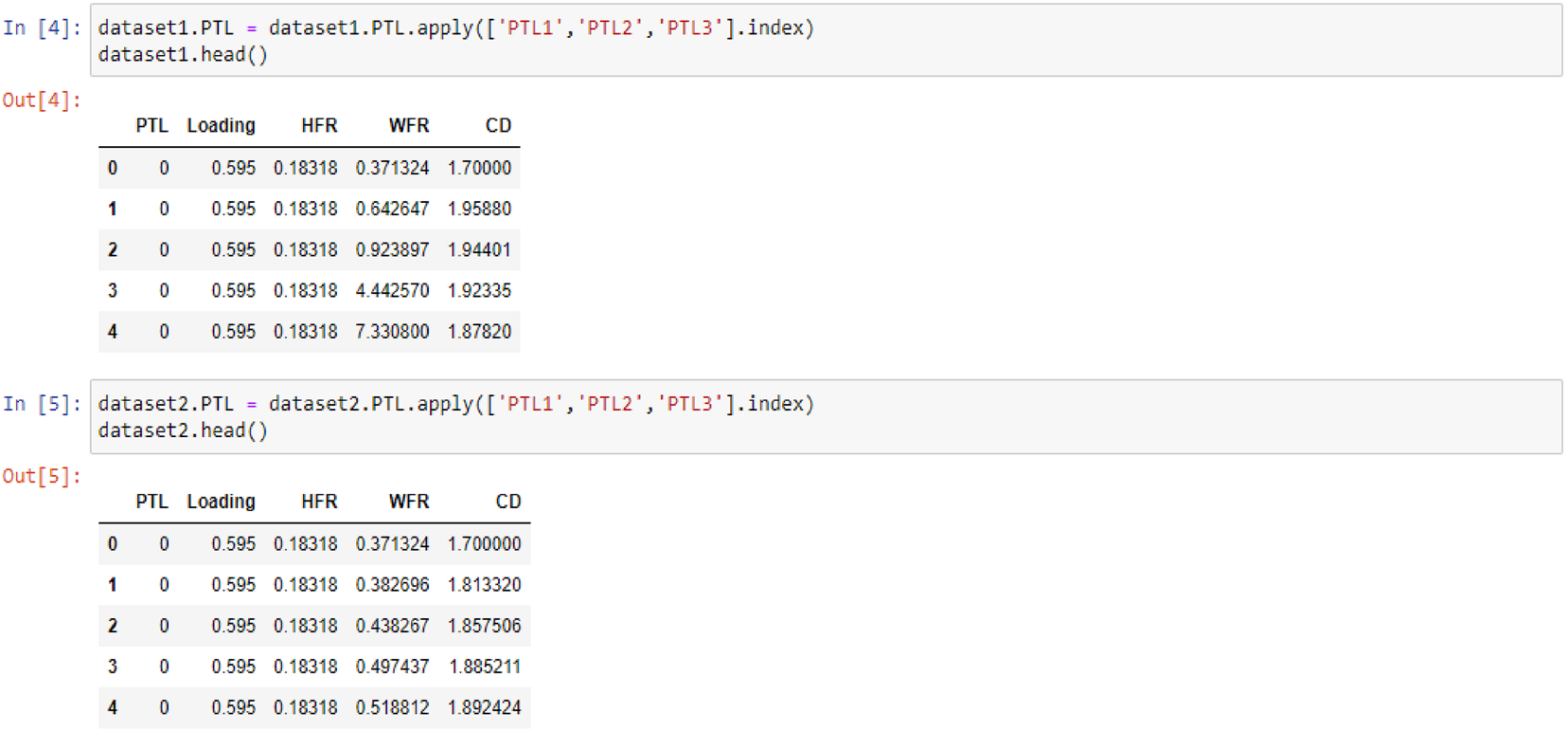


In the following, since all three selected models fall under the category of supervised learning, it is necessary to specify the features and target values for the analysis. To accomplish this, two variables, X and Y, are defined to represent the features and target values, respectively. Subsequently, the train and test data must be randomly selected from the dataset to enable the models to function optimally. In this study, 30% of the data were allocated to the test set, while 70% of the data were designated as the training set. The following codes can be used to implement the above:
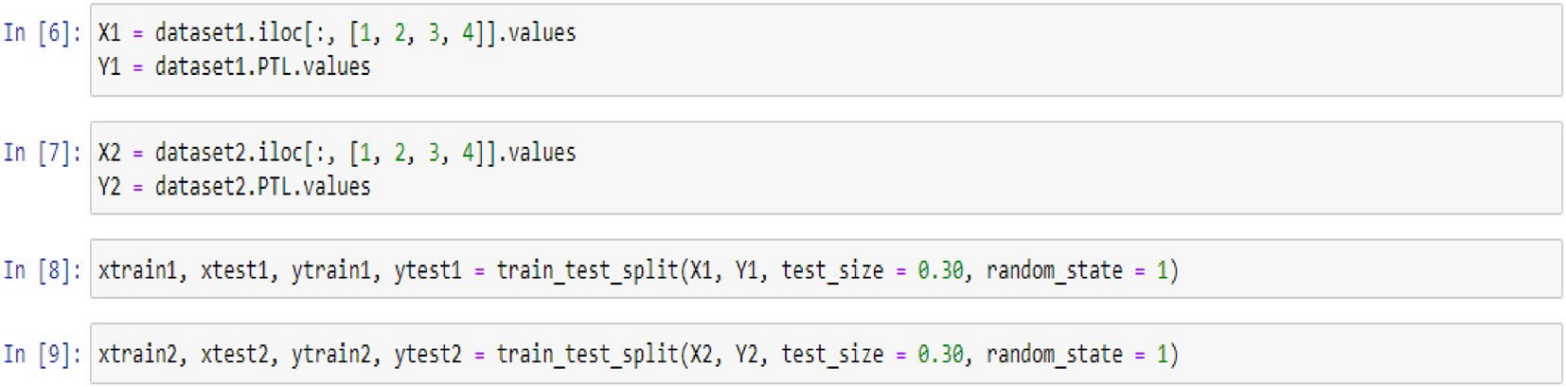


The acquired dataset encompasses features of diverse dimensions, collectively exerting a detrimental influence on the modeling of datasets, particularly in terms of accuracy rates, among other factors. Consequently, prior to executing the models, it is imperative to standardize the feature values utilizing the ensuing code. This procedure aims to adjust appropriately scaled dimensions conducive to effective model training.
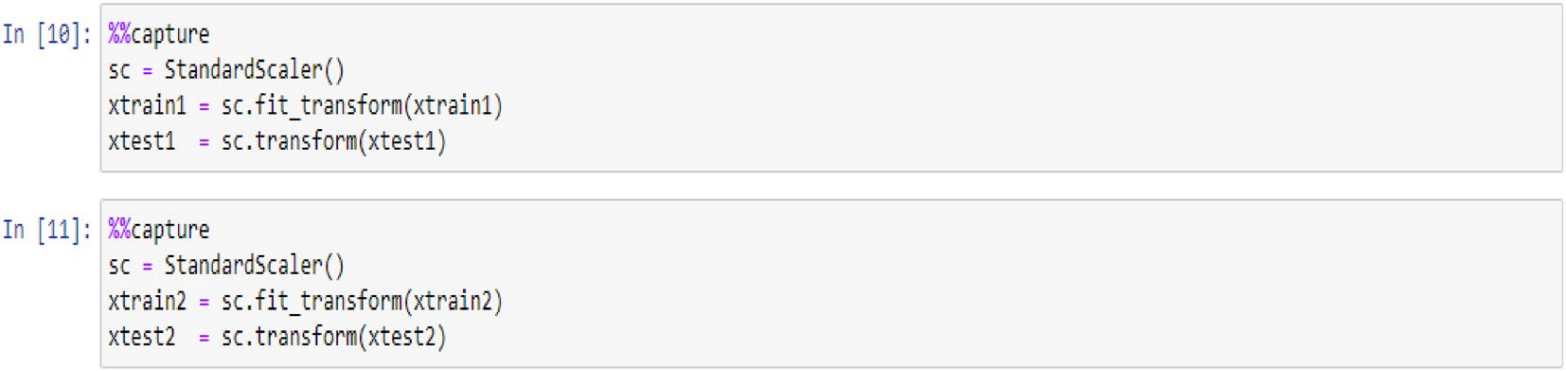


## Machine learning algorithms

In the following, the theory related to each of these models will be briefly explained and how to set them up along with the corresponding codes will be displayed:

### Regression model

Linear regression is one of the simplest supervised learning algorithms in our toolkit. If you have ever taken an introductory statistics course in college, likely the final topic you covered was linear regression. In fact, it is so simple that it is sometimes not considered machine learning at all! Whatever you believe, the fact is that linear regression and its extensions continues to be a common and useful method of making predictions when the target vector is a quantitative value (e.g., home price, age)^71^.

In this section, creating and fitting the Linear Regression model is explained which we can find the linear relationship between features and target vector besides the codes related to the prediction of the target values from the test features values:
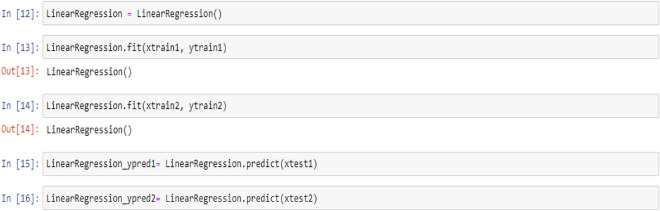


### Support vector machine (SVM) model

To comprehend support vector machines, it is helpful to first understand hyperplanes. In mathematical terms, a hyperplane refers to an (n-1) dimensional subspace within an n-dimensional space. Despite sounding complex, the concept is relatively simple. For instance, in a two-dimensional space, we could use a one-dimensional hyperplane (i.e., a line) to divide it. Conversely, in a three-dimensional space, a two-dimensional hyperplane (i.e., a flat plane or sheet) would suffice. In essence, a hyperplane is a generalization of this concept into n dimensions^71^. Support vector machines classify data by identifying the hyperplane that maximizes the margin between classes in the training data. In a two-dimensional example with two classes, the hyperplane is the widest straight "band" (i.e., line with margins) that separates the classes^71^.

In this section, we explain the process of building and training a Support Vector Machine (SVM) model, which seeks to identify the hyperplane that maximizes the margin between classes in the training data. We also provide the code employed to predict target values from test feature values.
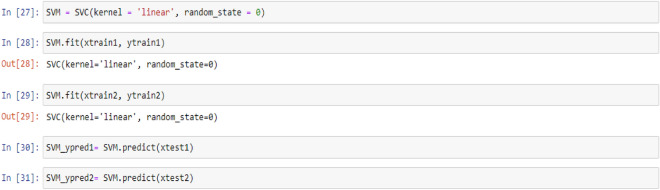


### Decision tree classifier model

Tree-based learning algorithms are a broad and popular family of related nonparametric, supervised methods for both classification and regression. The basis of tree-based learners is the decision tree wherein a series of decision rules (e.g., “If their gender is male…”) are chained. The result looks vaguely like an upside-down tree, with the first decision rule at the top and subsequent decision rules spreading out below. In a decision tree, every decision rule occurs at a decision node, with the rule creating branches leading to new nodes. A branch without a decision rule at the end is called a leaf^71^.

One of the primary reasons for the widespread adoption of tree-based models is their interpretability. Decision trees can be visually depicted in their entirety, thus enabling the creation of an intuitive model. This simple tree structure has spawned numerous extensions, ranging from random forests to stacking techniques^71^.

In this section, the process of building and training a Decision Tree Classifier model with a maximum unit depth of 1, 2, 3, and 4 were described. This model allows us to identify decision rules based on a non-parametric relationship between features and the target vector. Furthermore, the code used to predict target values from test feature values were provided as:

## Decision tree model (max-depth = 1)


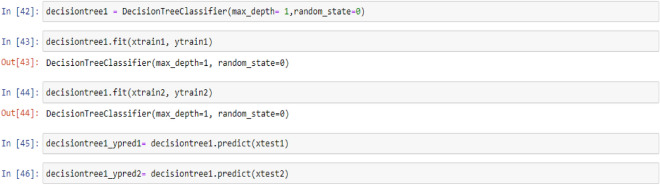


## Decision tree model (max-depth = 2)


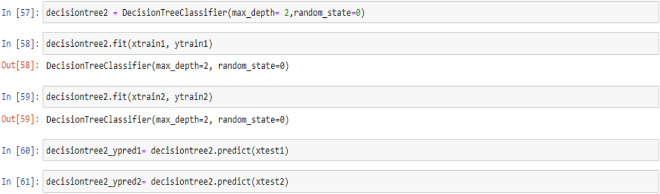


## Decision tree model (max-depth = 3)


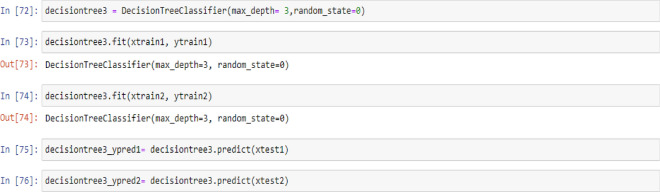


## Decision tree model (max-depth = 4)


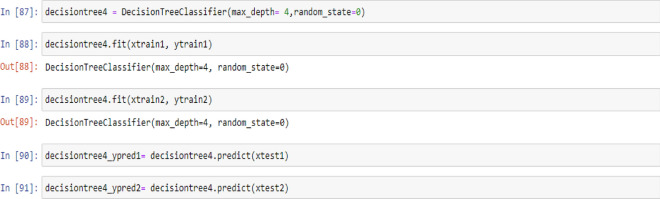


### Model evaluation and visualization

**Figure 3:**
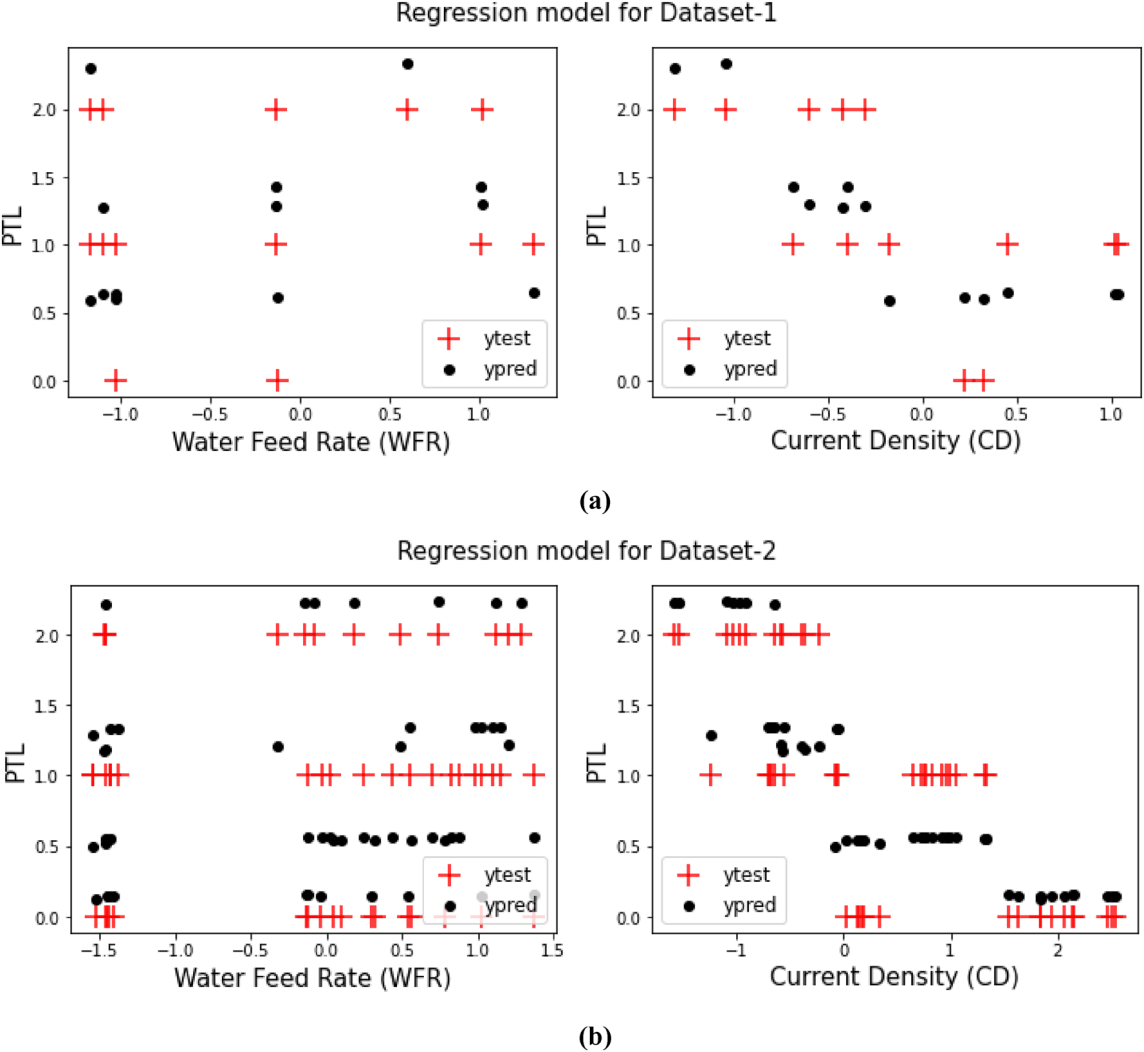
PTL vs. water feed rate (WFR) and current density (CD) for regression model (**a**) Dataset-1 (**b**) Dataset-2.

**Figure 4:**
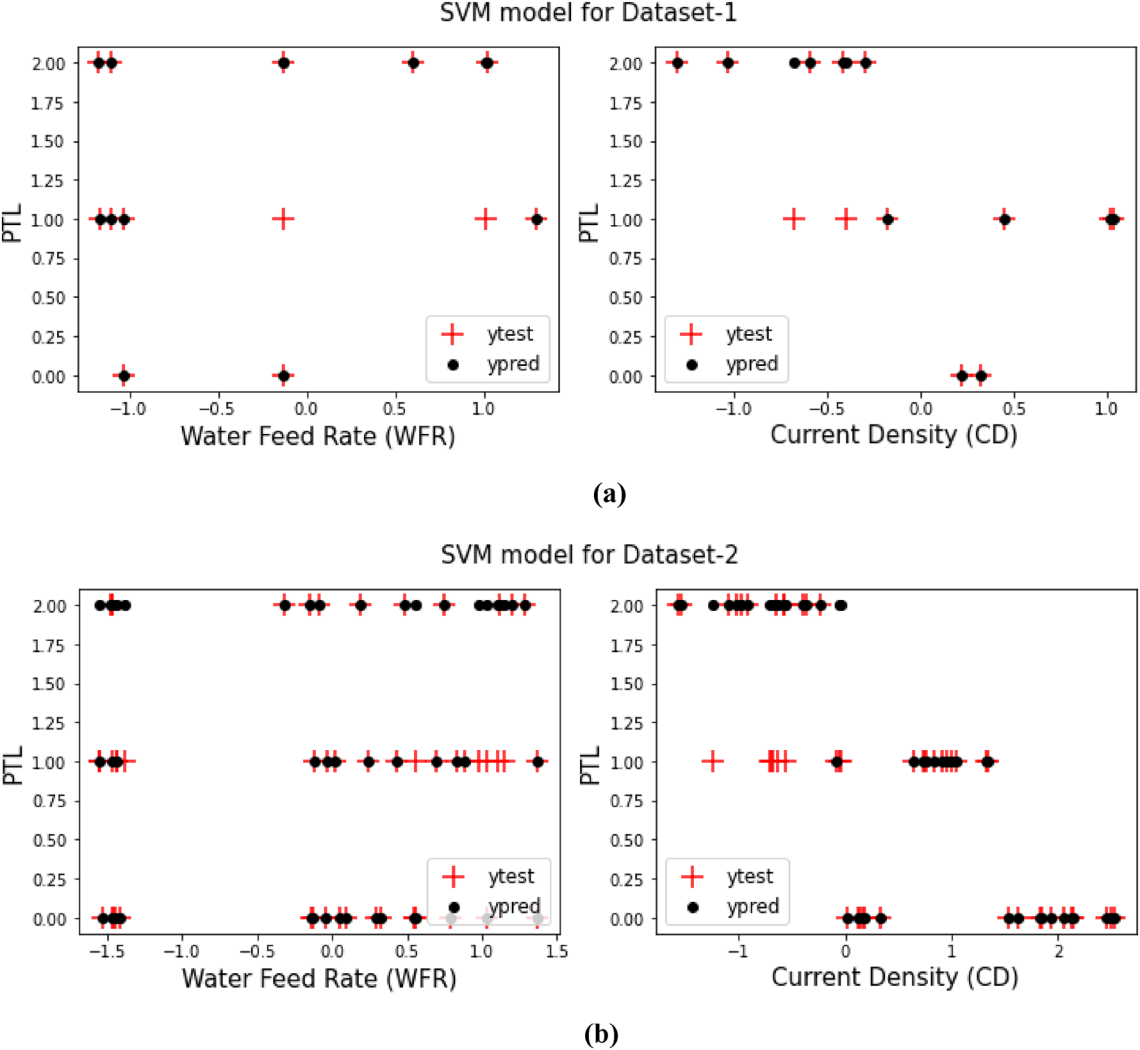
PTL vs. water feed rate (WFR) and current density (CD) for SVM model (**a**) Dataset-1 (**b**) Dataset-2.

**Figure 5:**
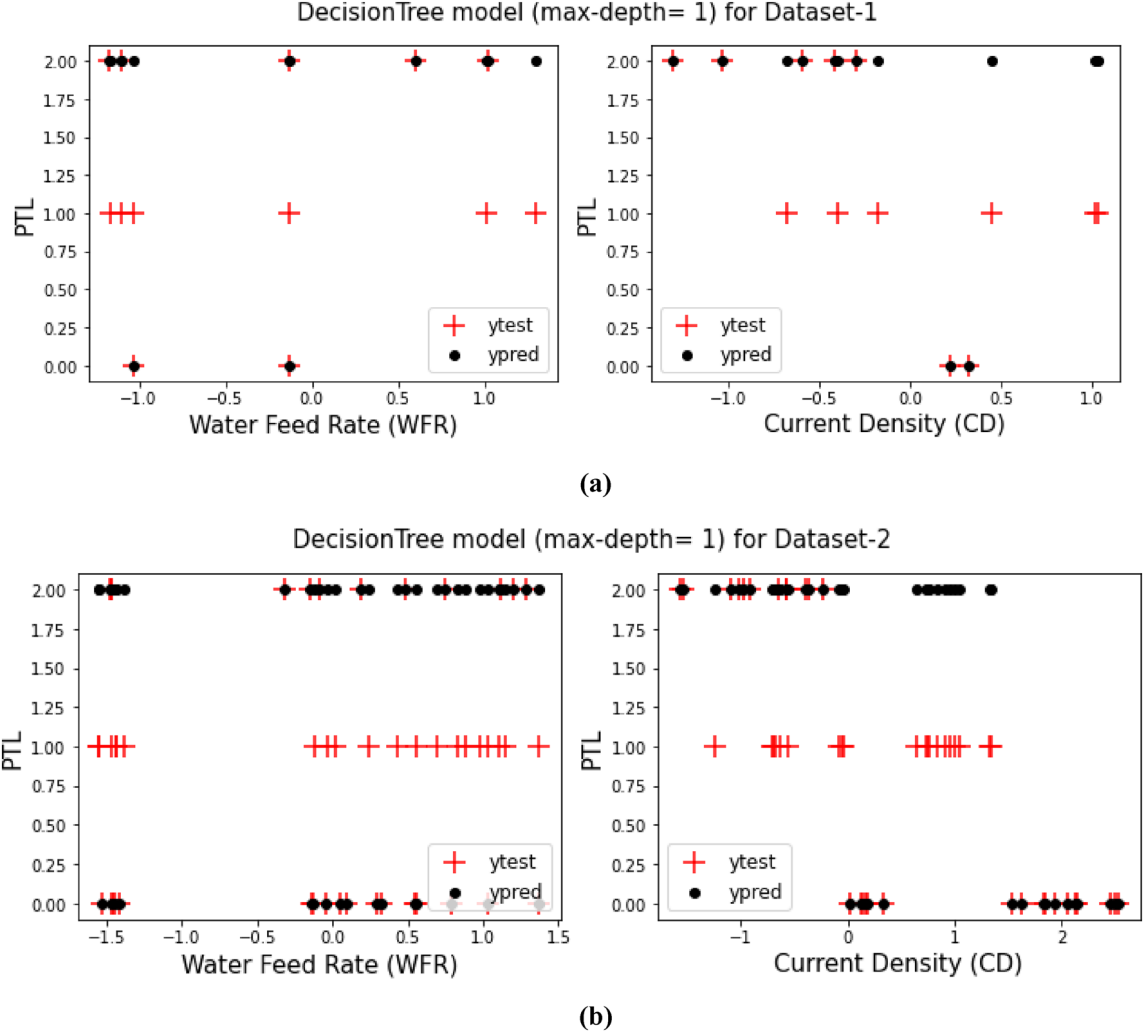
PTL vs. water feed rate (WFR) and current density (CD) for DecisionTree (max-depth = 1) model (**a**) Dataset-1 (**b**) Dataset-2.

**Figure 6:**
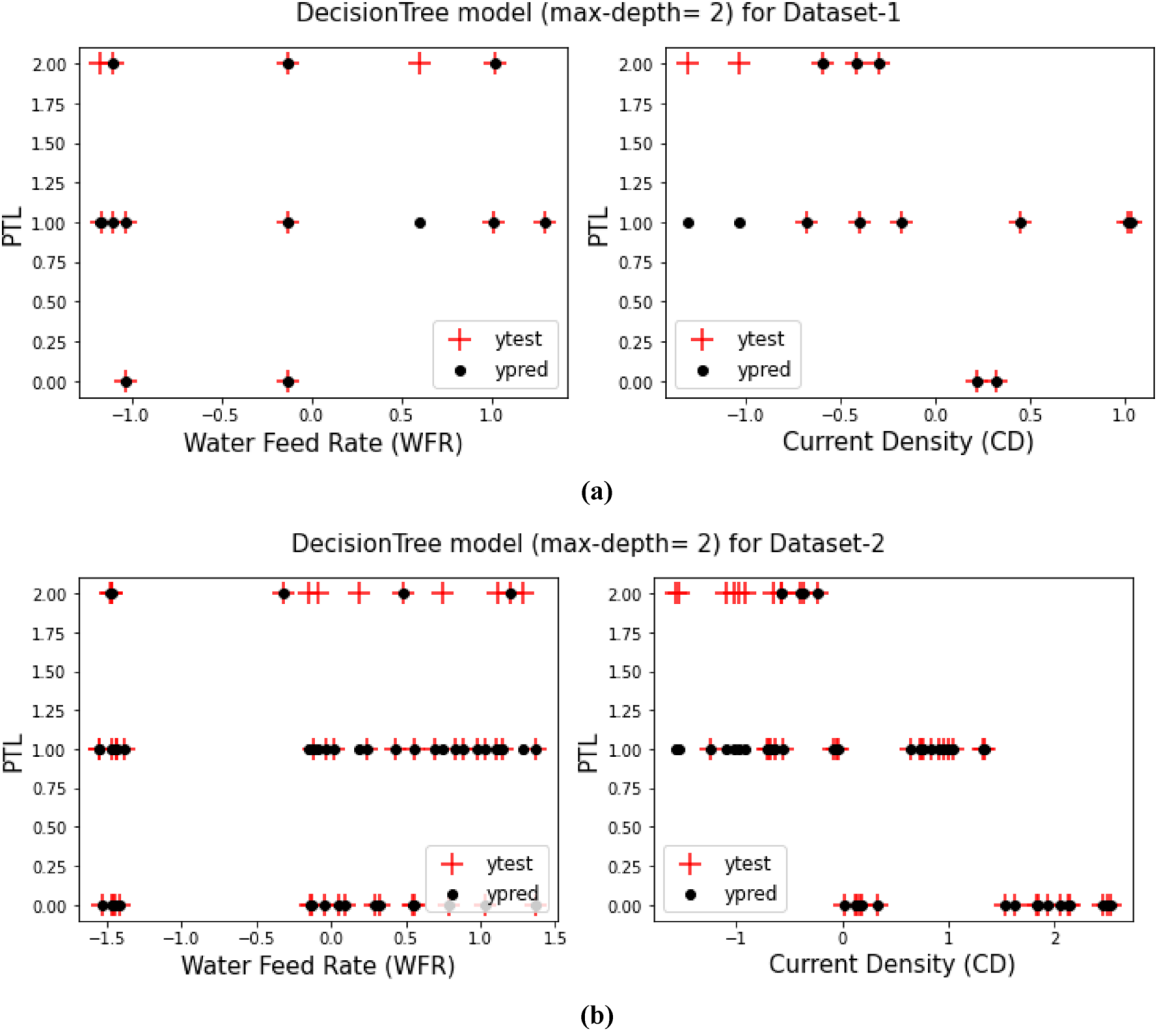
PTL vs. water feed rate (WFR) and current density (CD) for DecisionTree (max-depth = 2) model (**a**) Dataset-1 (**b**) Dataset-2.

**Figure 7:**
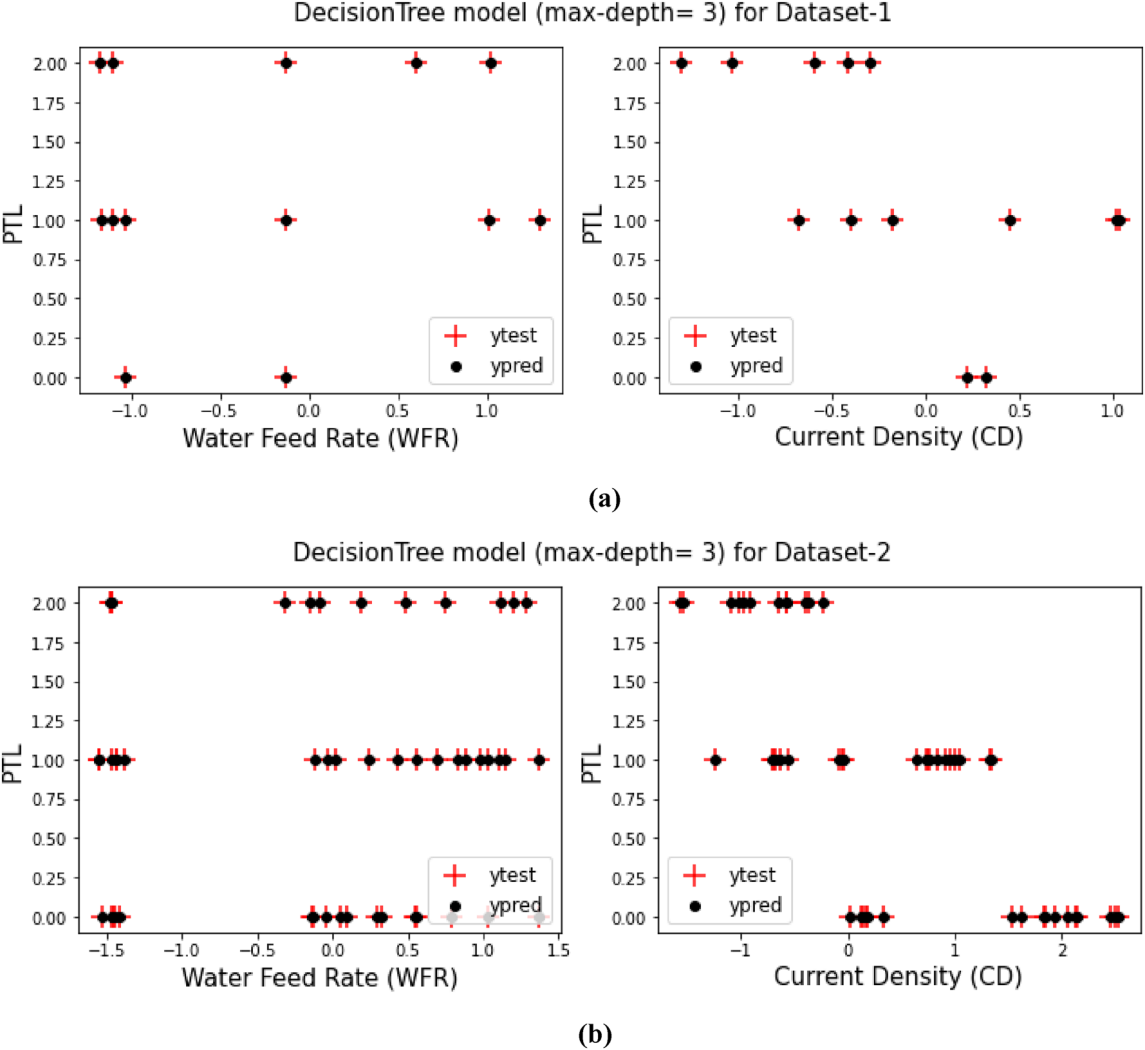
PTL vs. water feed rate (WFR) and current density (CD) for DecisionTree (max-depth = 3) model (**a**) Dataset-1 (**b**) Dataset-2.

**Figure 8:**
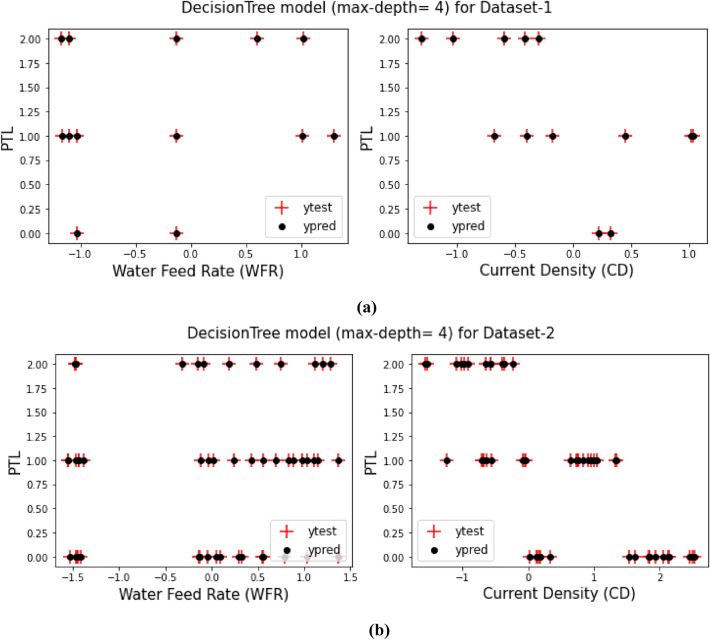
PTL vs. water feed rate (WFR) and current density (CD) for DecisionTree (max-depth = 4) model (**a**) Dataset-1 (**b**) Dataset-2.

As it said in previous sections, a comparison between the prediction target values from training the models and the test target values for both Dataset-1 and Dataset-2 have been displays in Figs. 3, 4, 5, 6, 7 and 8 based on the distribution of WFR and CD vs. PTL cases.

To check the efficiency of the models, there are number of parameters (Metrics), including model score (Accuracy), mean absolute error (MAE), mean squared error (MSE) and R^2^, which can be used to comparing the values of these parameters separately for each of the models using the following codes:
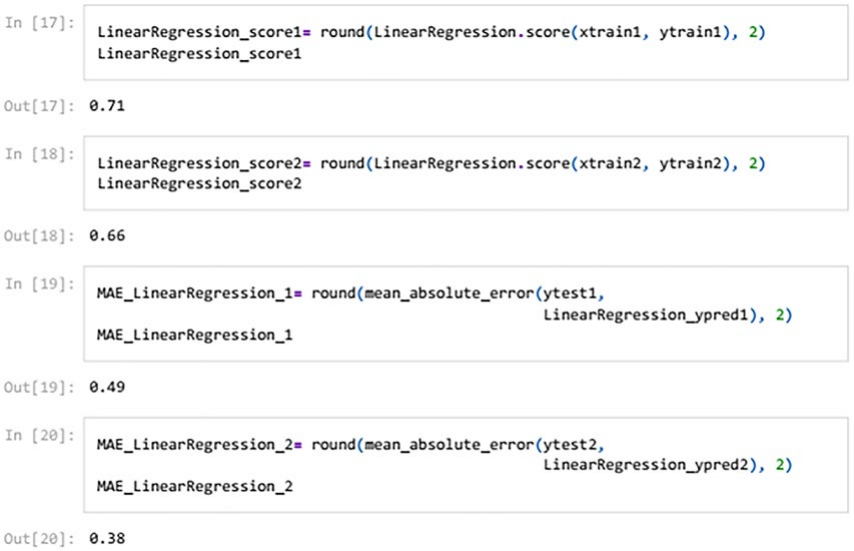

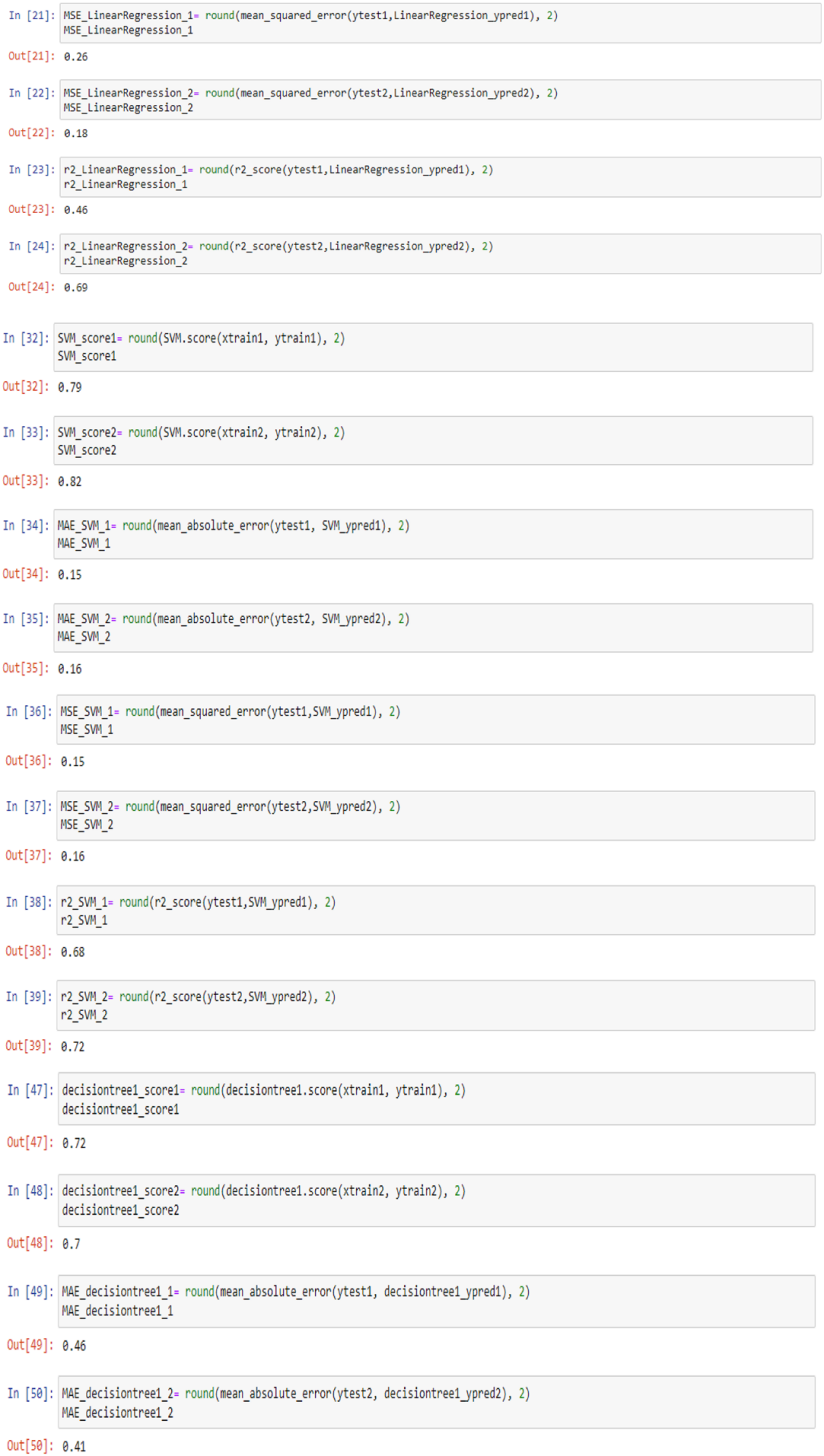

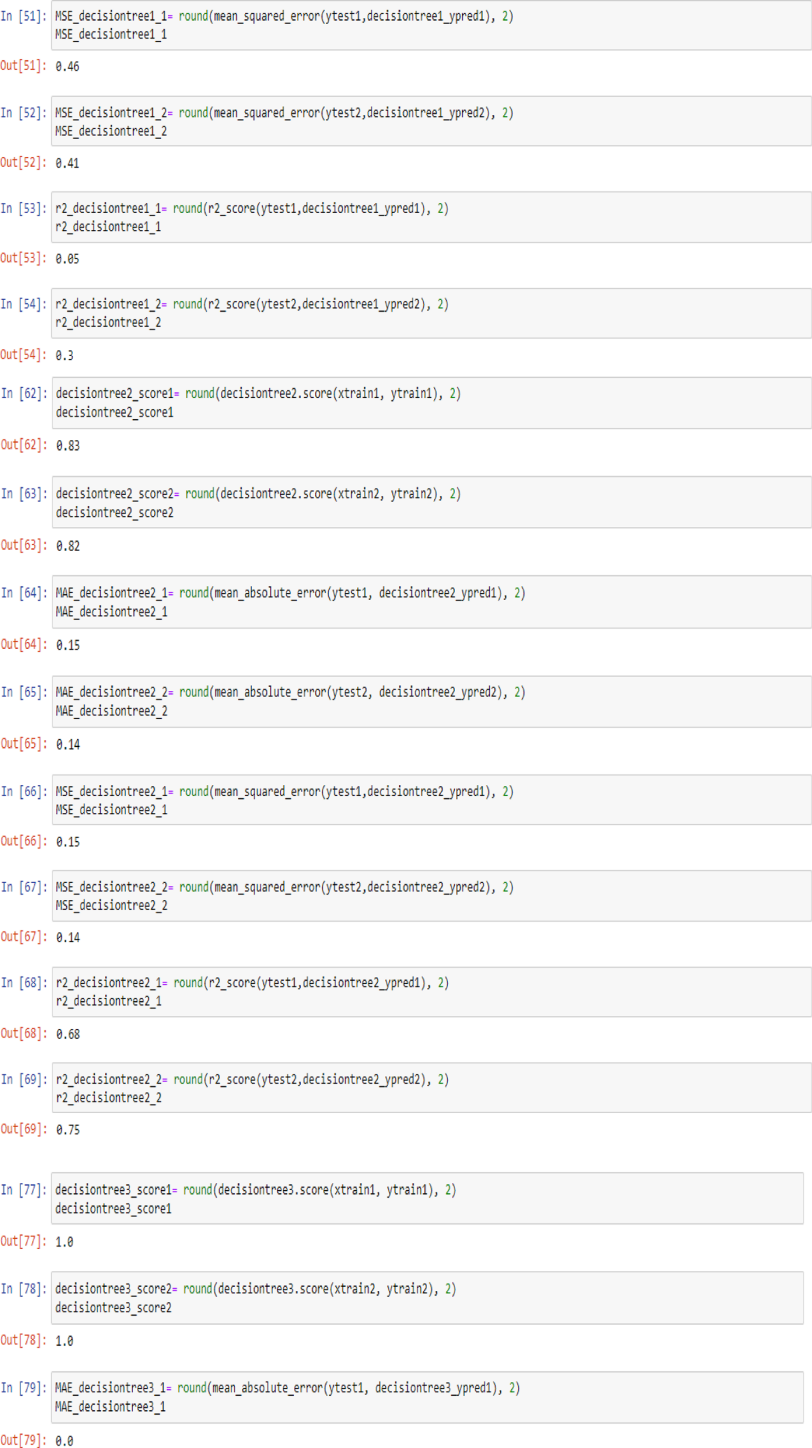

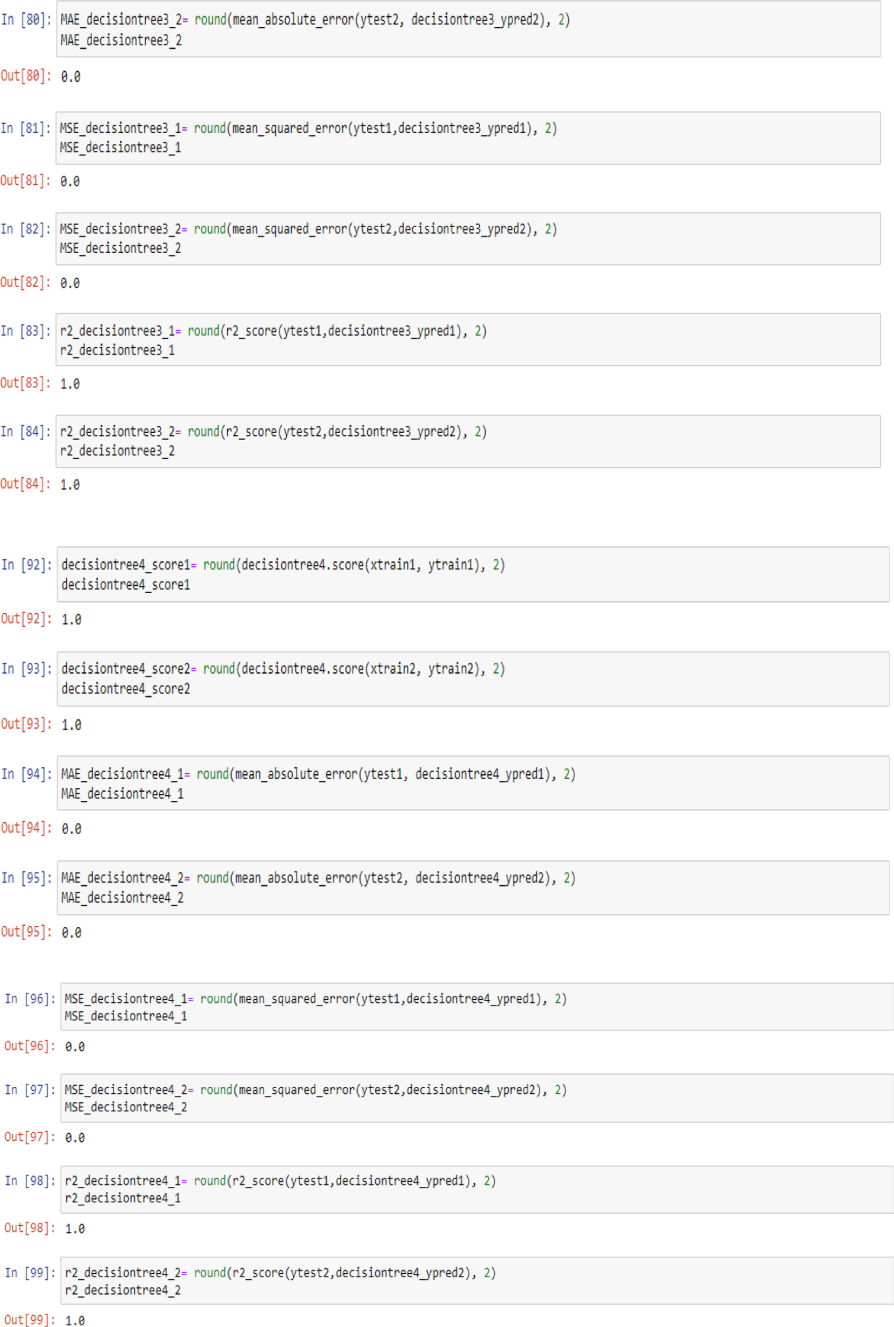


**Figure 9:**
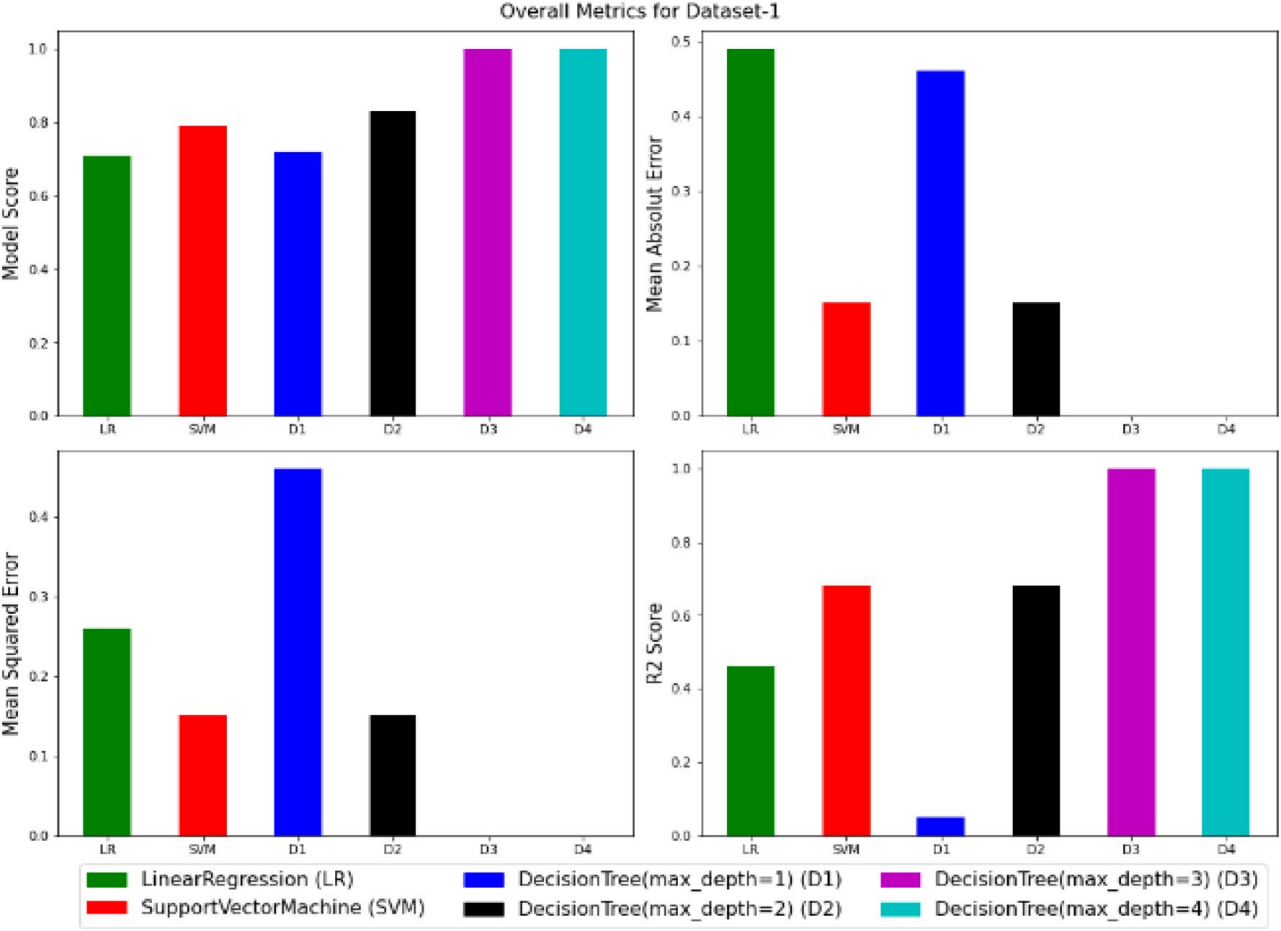
Overall metrics for Dataset-1.

**Figure 10:**
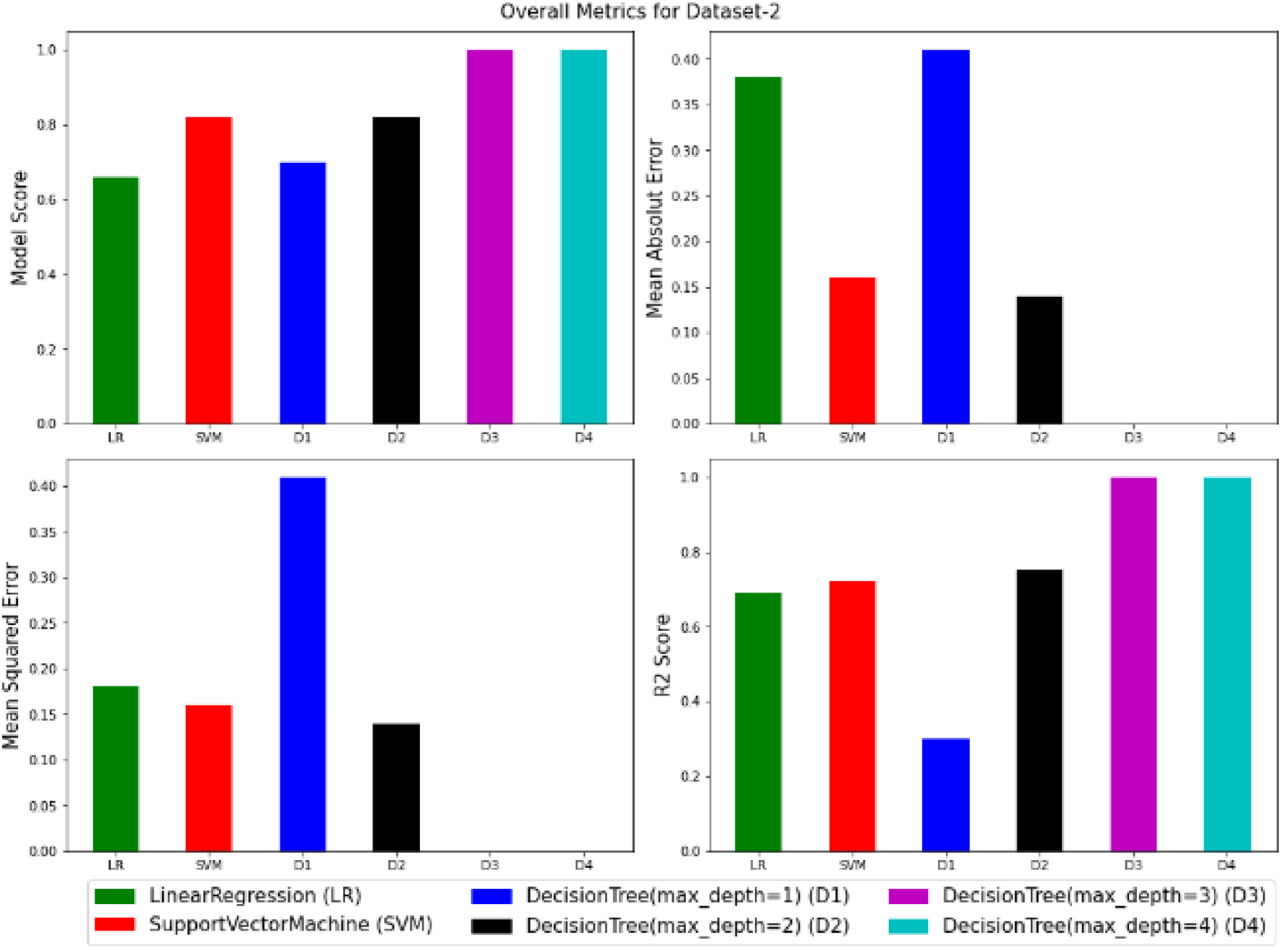
Overall metrics for Dataset-2.

To visualize the performance of the models, a comparison has been made between all three models on datasets 1 and 2 through the following codes and the values of model score (accuracy), mean absolute error (MAE), mean squared error (MSE) and R^2^ are displayed in Figs. 9 and 10 as the result of this work and the exact values of mentioned metrics have been tabulated in the Table 2.
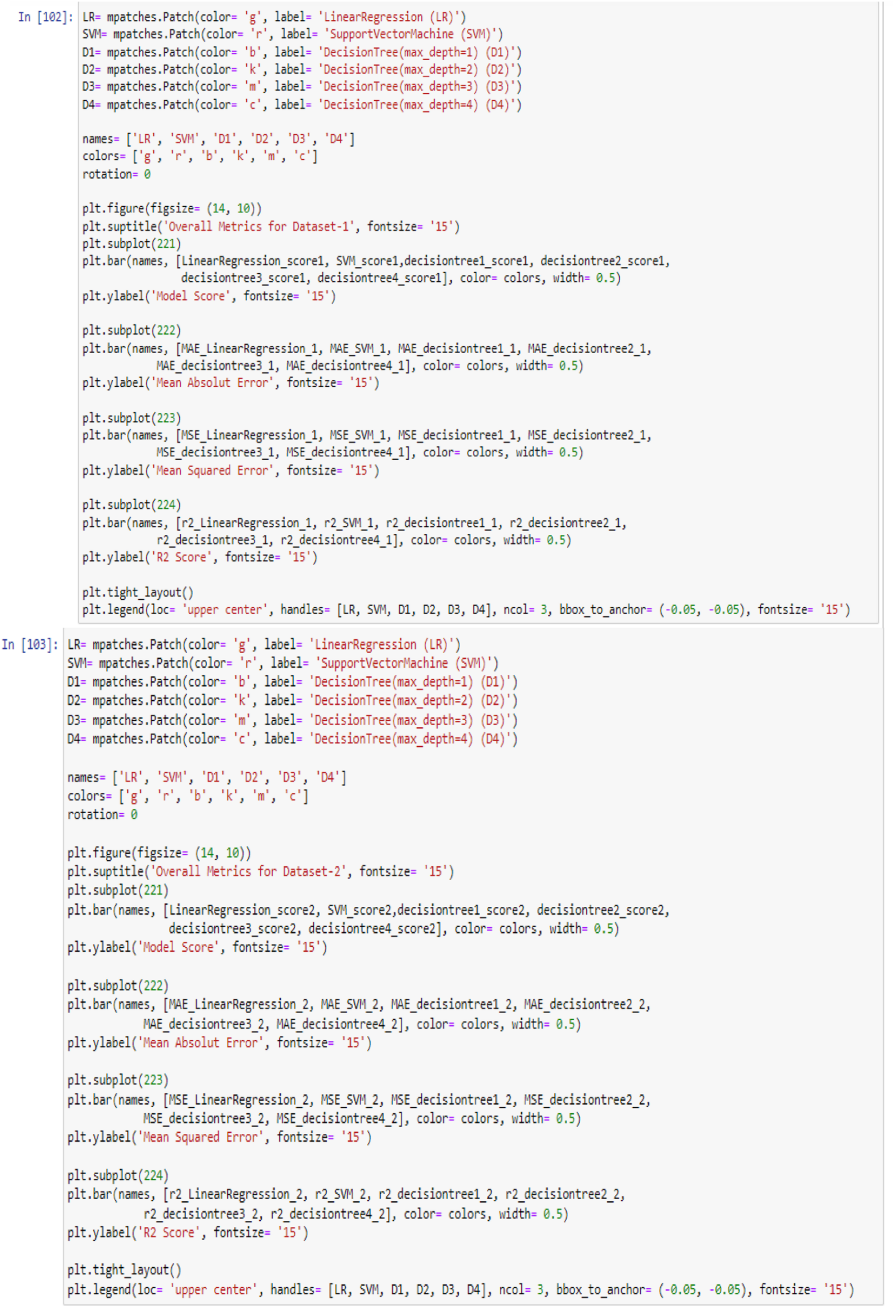

**Table 2:** Overall metrics values of models for Dataset-1 and Dataset-2.

Models	Overall metric values							
	Dataset-1				Dataset-2			
	Accuracy	MAE	MSE	R2-score	Accuracy	MAE	MSE	R2-score
Linear regression	0.71	0.49	0.26	0.46	0.66	0.38	0.18	0.69
Support vector machine	0.79	0.15	0.15	0.68	0.82	0.16	0.16	0.72
Decision tree (max-depth = 1)	0.72	0.46	0.46	0.05	0.70	0.41	0.41	0.3
Decision tree (max-depth = 2)	0.83	0.15	0.15	0.68	0.82	0.14	0.14	0.75
Decision tree (max-depth = 3)	1	0	0	1	1	0	0	1
Decision tree (max-depth = 4)	1	0	0	1	1	0	0	1

Figure 3 visually demonstrates that the regression model lacks accuracy and suffers from considerable error in classifying PTL types due to the incongruity between the test data (y_test_) and predicted data (y_pred_). As evident from Table 2, the model achieves an accuracy of only 71% with mean absolute error (MAE), mean squared error (MSE) and R^2^ of 0.49, 0.26 and 0.46, respectively. When the number of datasets increases, the accuracy declines further to 66% whereas MAE, MSE and R^2^ alter to 0.38, 0.18 and 0.69, respectively. Thus, the regression model is deemed unsuitable for monitoring the system’s behaviour.

Figure 4 illustrates the changes in WFR and CD for diverse PTL cases using the SVM model, ostensibly indicating that this model produces fewer prediction errors relative to the preceding model. As delineated in Table 2, the accuracy is 79% and 82% for datasets 1 and 2, respectively. The predictions from this model closely mirror the test data, thereby making the SVM model more fitting for modelling and anticipating system behaviour based on diverse parameters.

This work utilized decision tree models with maximum depths of 1, 2, 3 and 4 for modeling. Figures 5, 6, 7 and 8 illustrate the changes in WFR and CD based on PTL cases for these models. Figure 5 shows that the max-depth 1 model fails to predict PTL1 data due to its shallow structure as a result of tree pruning to a depth of one which makes the model to consider only one layer of datasets^71^. The metrics in Table 2 indicate this model is unsuitable. Figure 6 depicts the WFR and CD distribution for the max-depth 2 model, achieving 83% and 82% accuracy for datasets 1 and 2, respectively (Table 2). Unlike the previous model, it predicts all PTL cases due to its depth which could considering two layer of datasets^71^, making it suitable for predicting system changes and behavior. Figures 7 and 8 show the distributions for max-depth 3 and 4 models, respectively. The test and predicted data match perfectly, indicating highly accurate performance. Table 2 elucidates that their accuracy attains a remarkable 100% for both datasets, demonstrating exemplary model training and aptitude for predicting system behavior. The more intricate structures encompass all strata of data, culminating in flawless concordance between test and predicted data. To deepen our understanding, it is imperative to recognize that in decision tree models, the target value Y is forecasted by taking into account all input feature values denoted as X_1_, X_2_, …, X_P_, where P signifies the number of feature values or data layers. In this scenario, a binary tree is cultivated wherein, at each node, a test is conducted on one of the inputs or layers, denoted as X_i_. It is worth noting that every maximum depth of the tree corresponds to the consideration of a singular layer or feature value, denoted as X_i_^72^. Consequently, in the case of PTL versus water feed rate (WFR) and current density (CD) for the Decision Tree models with maximum depths of 3 and 4, for both datasets, three and four layers of the datasets were taken into account as input values. The tree was pruned to these specified depths, resulting in elucidated models boasting an accuracy of 100%^71^. Figures 9 and 10 compare the overall metrics for all models and datasets individually, allowing model comparisons based on accuracy, MAE, MSE and R^2^.

In summation, the incongruity between the test and predicted data for the regression model and its inferior predictive capability renders it an inappropriate choice for identifying PTL types. In contrast, the SVM model, due to its higher accuracy, congruous test and predicted data and comparatively lower errors, likely constitutes a more desirable option for the specified purpose.

Moreover, the shallower max-depth 1 model fails while the max-depth 2 model performs reasonably well. However, the max-depth 3 and 4 models achieve perfect accuracy and metric scores, indicating their suitability for the task due to their ability to account for all data layers through their deeper structures.

## Conclusion

The integration of artificial intelligence and machine learning, with a particular focus on their ability to gather and analyze data, has become a logical and essential consideration for chemists and chemical engineers seeking to understand flow patterns, develop empirical models, and design and optimize various systems. Recent research has elucidated the crucial role of devoting significant time and resources to the various stages of data science, encompassing everything from mining and preprocessing to data generation, inspection, and visualization. Furthermore, this research has emphasized the importance of scientific expertise in leveraging the full potential of data science, particularly in the context of chemistry and chemical engineering. The data used in this study was obtained from open literature sources, with a primary focus on data science and machine learning. The project involved conducting a data science analysis on a set of data related to operational parameters affecting the anode side of a PEM water electrolyzer for H_2_ production, using various machine learning models and comparing their performance. One of the most important issues proven in this article is data generation, which clearly has a direct impact on the conclusion and analysis of the system by approaching overall metrics values to the reality. While some individuals may believe that increasing the volume of data will necessarily enhance the accuracy of models, this study demonstrates that as the number of data points increases, the models become more realistic, and the accuracy becomes more precise, even if it leads to a decrease in accuracy. As demonstrated in the text, the accuracy value for the SVM model increased after data generation, while it decreased for the regression, Decision Tree (max-depth = 1), and Decision Tree (max-depth = 2) models.

This endeavor delved into the exploration of data science and machine learning in conjunction with the hydrogen gas production via water electrolysis. It entailed an in-depth analysis of data pertaining to the anode side catalyst of PEMWE, with a particular emphasis on the criticality of data pre-processing, notably data generation. Consequently, it was substantiated that employing models for prognostication, analysis, and optimization profoundly impacts system efficiency. Presently, the substitution of non-renewable energy sources with renewable alternatives, as well as the development of novel, efficient, and cost-effective solutions for storing excess electricity generated by renewable systems like wind and solar, stands as a paramount challenge for humanity. This shift has profound implications for the future of our planet. Hence, we anticipate that this research, with its attendant benefits and forward-looking perspectives, will positively contribute to the advancement of hydrogen gas production as a clean fuel. Moreover, it holds the promise of informing the design of a storage system capable of accommodating excess electricity generated from renewable sources. This will be achieved through the meticulous analysis of data pertaining to the anode side of PEMWE and the derivation of a robust model for the precise prediction, analysis, and optimization of system efficiency. In conclusion, data science and machine learning provide valuable insights and can serve as useful tools for chemists and chemical engineers. They can expand their knowledge base and add to their toolbox.

## Data Availability

All data generated or analysed during this study are included in this published article.
